# Validity and Reliability of an Electronic Contact Mat for Drop Jump Assessment in Physically Active Adults

**DOI:** 10.3390/sports7050114

**Published:** 2019-05-16

**Authors:** Florian Tenelsen, Dennis Brueckner, Thomas Muehlbauer, Marco Hagen

**Affiliations:** Division of Movement and Training Sciences/Biomechanics of Sport, University of Duisburg-Essen, 45141 Essen, Germany; florian.tenelsen@stud.uni-due.de (F.T.); dennis.brueckner@uni-due.de (D.B.); thomas.muehlbauer@uni-due.de (T.M.)

**Keywords:** jump, force plate, ground contact time, lower-extremity muscle power

## Abstract

The aim of the present study was to investigate the concurrent validity and the test–retest reliability of an electronic contact mat for drop jump assessment in physically active adults. Seventy-nine young, physically active adults participated in the validity study, and 49 subjects were recruited for the reliability study. The motor task required subjects to perform two-legged drop jumps using drop heights of 24, 43, and 62 cm as well as one-legged drop jumps with the left and right leg using a drop height of 24 cm. Ground contact times were simultaneously quantified with an electronic contact mat, a force plate (i.e., gold standard), and a light-barrier system (another criterion device). Concurrent validity was assessed using intraclass correlation coefficient (ICC), systematic bias, limits of agreement, and linear regression analysis. Test–retest reliability (one week apart) was determined by calculating the ICC, the standard error of measurement (SEM), the coefficient of variation (CV), and Lin´s concordance correlation coefficient (р_c_). Further, we determined the minimal detectable change (MDC_95%_). Irrespective of drop height and jump condition, good agreements between testing devices (ICC ≥ 0.95) were shown. Compared to the force plate (−0.6 to 3.1 ms) but not to the light-barrier system (31.4 to 41.7 ms), the contact mat showed low systematic bias values. In terms of test–retest reliability, our analyses showed that the measuring devices are in agreement (ICC: 0.70–0.92; SEM: 8.5–18.4 ms; CV: 3.6–6.4%). Depending on the measurement device, drop height, and jump condition, a MDC_95%_ value ranging from 23.6 to 50.9 ms represents the minimum amount of change needed to identify practical relevant effects in repeated measurements of drop jump performance. Our findings indicate that the electronic contact mat is a valid and reliable testing device for drop jump assessment from different drop heights in young physically active adults.

## 1. Introduction

In game sports (e.g., soccer), but also in various disciplines of track and field, reactive movement behavior is an important component of athletic performance. For example, Gissis et al. [1] were able to show that young soccer players from the national squad achieved significantly larger drop jump heights than players on a regional and leisure level. In addition, Coh and Mackala [2] demonstrated significantly larger jump heights and take-off velocities, as well as lower ground contact times among successful top sprinters compared to less successful ones. In addition, ground contact time is an important parameter for estimating the efficiency of reactive movements in the stretch shortening cycle (SSC). Bosco et al. [3] were able to show that increased muscle activity increases leg stiffness during drop jumps and thus reduces ground contact time.

The assessment of reactive movement behavior is therefore important from a physical performance and training perspective in order to identify athletes with below-average (injury risk) or above-average (talent search) performance, as well as to document exercise-related changes in performance (training effectiveness). The gold standard for recording ground contact time is the use of a force plate, but its purchase is more expensive compared to a contact mat and therefore hardly feasible for sports clubs. Electronic contact mats are an inexpensive alternative, and these can also be transported with little effort and easily used under field conditions. Despite these advantages, contact mats must fulfil validity and reliability criteria in order to carry out an adequate analysis of the reactive movement behavior. In this regard, Kenny et al. [4] conducted a study with ten athletic young adults in which they analyzed the concurrent validity of an electronic contact mat versus a force plate for the drop jump (drop height: 30 cm). With regard to the ground contact time, only a small Pearson correlation coefficient (r = −0.17) was found between the two measuring devices. Another study by Rogan et al. [5] included ten young adults. The drop jump was conducted from a drop height of 40 cm and the ground contact time was recorded simultaneously with an electronic contact mat and a force plate. In contrast to the previous work, a high Pearson correlation coefficient (r = 0.98) was observed between the two test devices, which supports the use of the contact mat for a valid drop jump analysis. Although the aforementioned studies [4,5] extended the state of knowledge on the concurrent validity of contact mats in comparison to force plates for the investigation of the drop jump, the analyses revealed different findings and showed methodological limitations (e.g., inclusion of few subjects (N = 10); examination of a single drop height).

In addition to studies on concurrent validity, there are studies [6,7] which analyzed the test–retest reliability of an electronic contact mat for drop jump assessment. For example, Markwick et al. [6] examined physically active young adults (N = 13), who each completed three drop jumps at intervals of one minute from drop heights of 20, 30, 40, and 50 cm. Irrespective of the drop height, “excellent” correlations (intra-class correlation coefficient (ICC) = 0.87–0.99) were found between the tests, indicating that the contact mat can be used for the reliable analysis of drop jumps. However, it remains unclear whether this finding will be confirmed even at longer test intervals of several days. Even though, the present study focused on the assessment of drop jumps, there are further studies available that investigated concurrent validity and test–retest reliability of electronic contact mats by using an electric input circle [8] or squat and countermovement jumps [9]. These studies showed that contact mats provide trustworthy measurements for the valid and reliable assessment of vertical jumps.

Therefore, the aim of the present study was to analyze the concurrent validity and test–retest reliability of an electronic contact mat versus a force plate (gold standard), and a further criterion device (light-barrier system) for drop jump assessment. The investigation of (i) two- and one-legged drop jumps, (ii) different drop heights (i.e., 24, 43, and 62 cm), and (iii) a relatively large sample (N = 46–79) of young adults represent novel aspects and extends the findings of previous studies [4,5,6].

## 2. Materials and Methods

### 2.1. Participants

Seventy-nine young, physically active adults of both sexes took part in the validation study, and 46 of those subjects were additionally tested in the reliability study. Their age ranged between 20 to 30 years. All subjects had previous experience with carrying out the drop jump that was acquired in study courses prior to their assessments. None of the subjects had musculoskeletal, neurological or orthopedic impairment of the lower extremities. All subjects were informed about the contents of the respective study and gave their written consent to participate. The protocol of the study was approved by The Human Ethics Committee at the University of Duisburg-Essen, Germany (TM_29.11.2018).

### 2.2. Jump Task

The motor task was the two- and one-legged drop jump. During the jump, the participants stood at hip’s width on a height-adjustable box. Their hands were on their hips. On the verbal command “Ready! Go!” they jumped with one leg. After landing, the subjects had to jump off the ground explosively. The corresponding instruction was: “Jump up from the ground as fast and as high as possible after landing”. The body remained stretched during the flight phase.

### 2.3. Experimental Setup

**Figure 1:**
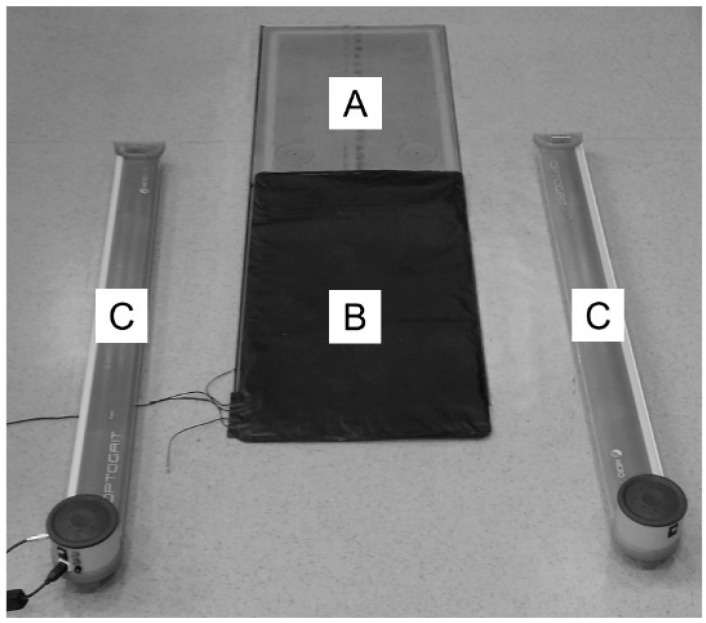
Experimental setup consisting of a force plate (**A**), an electronic contact mat (**B**) above the force plate, and an adjacent light-barrier system (**C**) for the simultaneous and synchronized (sample frequency: 1000 Hz) recording of ground contact time.

The required ground contact time was simultaneously recorded by three measuring devices (Figure 1). This involved a force plate (Kistler^®^, type 9281b, dimensions (mm): 600 × 400 × 100, Winterthur, Switzerland), the OptoGait^®^ light-barrier system (Microgate, Bolzano, Italy), and an electronic contact mat (Conrad Electronic SE, type 751913, dimensions (mm): 710 × 405 × 6, Hirschau, Germany). The synchronized recording using a sampling frequency of 1000 Hz as well as the numerical and graphical representation of the ground contact time was carried out with the LabVIEW software (National Instruments, Austin, TX, USA).

### 2.4. Test Procedure

**Figure 2:**
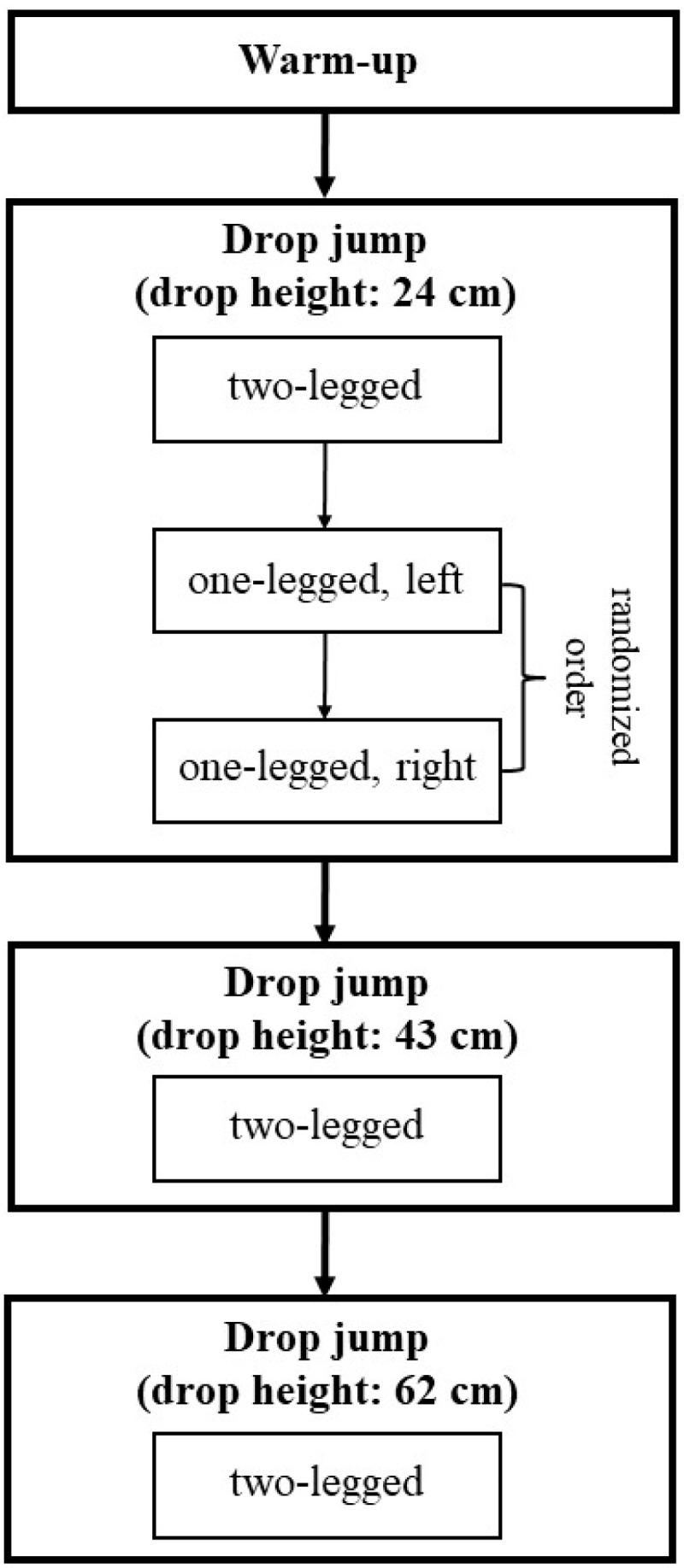
Schematic diagram of the testing procedure.

At the beginning of the test, each subject received a verbal instruction and a practical demonstration of the test procedure (Figure 2). This was followed by a general warm-up (1200 m run at a moderate pace) followed by a specific warm-up (bouncing on both legs and one leg, as well as drop jumps from a low drop height). Afterwards, drop jumps from a drop height of 24 cm were performed, first two-legged and then one-legged in a randomized order (left/right leg). Subsequently, two-legged drop jumps were performed from drop heights of 43 cm and 62 cm. Each jump condition included one practice trial and three data-registration trials. The mean ground contact time (ms) from the three data-registration trials was determined as the dependent variable. The rest between the jump trials was 30 seconds and, between the jump conditions, one minute. The participants of the validation study carried out the test once, and the subjects of the reliability study twice, at intervals of one week.

### 2.5. Statistical Analysis

To describe the test results, the mean value was calculated as a measure of the central tendency and the standard deviation (SD) as a dispersion measure. The concurrent validity was analyzed by calculating the ICC_2,1_ (i.e., two-way random-effects model) with a 95% confidence interval (CI) because we randomly selected the rater from a larger population of raters with similar characteristics (i.e., graduate sports scientists) [10]. Bland–Altman graphics [11] have been created to illustrate the agreement between the measuring devices. As a result, 95% of the data points should be within the limits of agreement (i.e., mean value ± 1.96 SD) [11]. Further, the systematic bias (i.e., mean difference) and the width of the limits of agreement (LoA) were calculated [12]. Lastly, a linear regression analysis was conducted to see whether the intercept and slope differed from 0 and 1, respectively. The analysis of the relative reliability was conducted on the basis of ICC_3,1_ (i.e., two-way mixed-effects model) with a 95% CI [13], and classified according to Fleiss [14] as an "excellent" (ICC ≥ 0.75), a "moderate to good" (0.40 < ICC < 0.75) or a "worse" (ICC ≤ 0.40) correlation. The two-way mixed-effects model was chosen because the results only represent the reliability of a specific rater that was responsible for the reliability experiment [10]. In addition, Lin´s concordance correlation coefficient (р_c_) was calculated [15]. The absolute reliability was determined by calculating the standard measurement error (SEM) [13]. For the SEM value, it is postulated that the smaller the value, the higher the reliability of repeated measurements. Additionally, the coefficient of variation (CV) was calculated. According to Stokes [16], CV values of ≤15% can be classified as satisfactory. Furthermore, the minimum detectable change (MDC_95%_) was calculated [13,17]. The MDC_95%_ value indicates how high the change between repeated measurements must be in order to exclude a measurement error and detect a real change. The statistical analyses were carried out with the software Statistical Package for Social Sciences, version 24.0 (IBM, Armonk, NY, USA).

## 3. Results

The results obtained in the first and second assessment are given according to the measuring instrument in Table 1. Although there were some statistical differences between means, the corresponding effect size values were solely small (i.e., *d* ≤ 0.49). Irrespective of the jump condition, there were fewer deviations in the achieved ground contact times between the electronic contact mat and the force plate (~1–4 ms) than between the contact mat and the OptoGait^®^ light-barrier system (~30–43 ms).

### 3.1. Concurrent Validity

**Figure 3:**
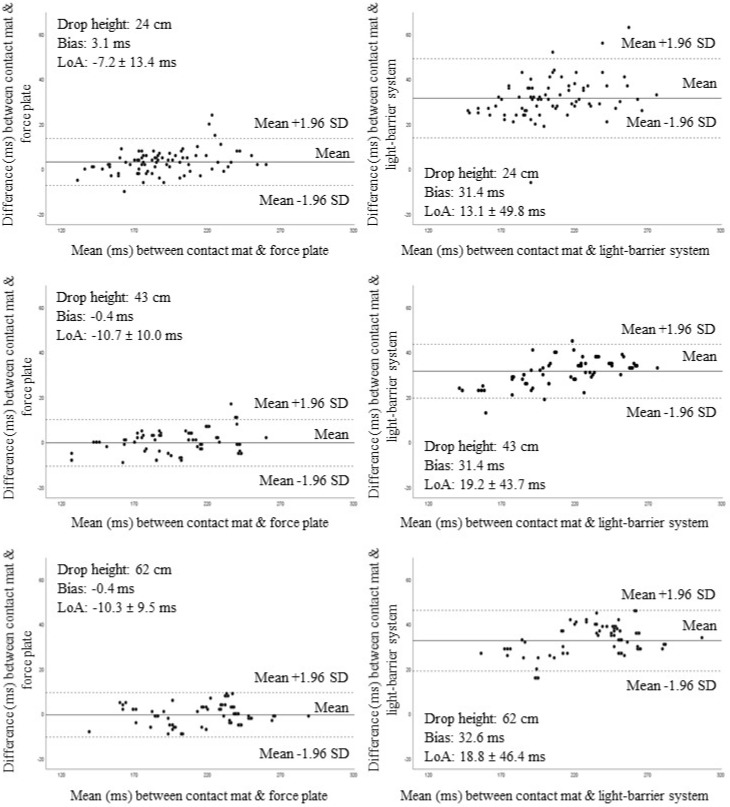
Bland–Altman plots for the comparison of the electronic contact mat with the force plate (corresponds to the gold standard) (see left-hand side) and the OptoGait^®^ light-barrier system (further criterion device) (see right-hand side) for the two-legged drop jump from drop heights of 24, 43, and 62 cm. The dashed lines indicate the limits of agreement (LoA) within which 95% of the data points should be located (corresponds to the mean ± 1.96 standard deviations (SD)).

The comparison of the electronic contact mat with the force plate showed only “excellent” ICC values of ≥0.95, independent of drop height and jump condition (Table 2). Furthermore, the Bland–Altman plots revealed that only 4 of 79 values (5.1%), 3 of 79 values (3.8%), and 0 of 79 values (0%) were outside the agreement limits when the drop jump was carried out two-legged from drop heights of 24, 43, and 62 cm (Figure 3, left side). Values for the systematic bias and the corresponding LoA are displayed in Table 2. In addition, the regression analyses revealed that the slope significantly differed from 1 and that the coefficient of determination (R^2^) ranged between 0.91 and 0.98. This indicates that data from the contact mat explained 91–98% of the variance of data from the force plate. The comparative examination of the contact mat with the light-barrier system yielded ICC values of 0.95 to 0.98, which demonstrate "excellent" relationships. The Bland–Altman plots showed that only 4 of 79 values (5.1%), 3 of 79 values (3.8%), and 3 of 79 values (3.8%) were outside the agreement limits when the drop jump was performed two-legged from drop heights of 24, 43, and 62 cm (Figure 3, right side). Further, the values for the systematic bias and the corresponding LoA are shown in Table 2. With respect to the regression analyses, both the intercept and the slope significantly differed from 0 and 1, respectively. The R^2^ values ranged between 0.91 and 0.98, which means that data from the contact mat explained 91–98% of the variance of data from the OptoGait^®^ system.

### 3.2. Test–Retest Reliability

For the two-legged jumping conditions, only “excellent” ICC values (0.75–0.90) were found, independent of the measuring device (Table 3). In the one-legged jumping conditions, “excellent” ICC values (0.84–0.92) were found in the majority of cases and “moderate to good” ICC values (0.70–0.74) in a few cases. The SEM was in a range from 10.3 to 18.4 ms for the force plate, from 10.6 to 18.1 ms for the OptoGait^®^ light-barrier system, and from 8.5 to 17.0 ms for the electronic contact mat, depending on the jump condition (Table 3). In addition, CV values were below the threshold of ≤15% and ranged from 3.6% to 6.4%, irrespective of measurement device, drop height, and jump condition (Table 3). Lastly, the р_c_ value ranged from 0.69 to 0.90 for the force plate, from 0.74 to 0.89 for the OptoGait^®^ light-barrier system, and from 0.70 to 0.88 for the contact mat (Table 3).

### 3.3. Minimal Detectable Change

The MDC_95%_ values were in a range from 28.4 to 50.9 ms for the force plate, from 29.4 to 50.1 ms for the OptoGait^®^ light-barrier system, and from 23.6 to 47.0 ms for the electronic contact mat, depending on the jump conditions (Table 4).

## 4. Discussion

The present study analyzed the concurrent validity and the test–retest reliability of an electronic contact mat in comparison to a force plate (gold standard) and a further criterion device (light-barrier system) for recording ground contact time during the execution of drop jumps from different drop heights by young, physically active adults. The results can be summarized as follows: (i) exclusively “excellent” ICC values (≥0.95) related to the analysis of the diagnostic validity of the contact mat versus the force plate or the light barrier-system, (ii) the contact mat showed rather low systematic bias values when compared to the force plate (–0.6–3.1 ms), but not when compared to the light-barrier system (31.4–41.7 ms), (iii) “moderate to good” to “excellent” ICC values (0.70–0.92) with respect to relative reliability, (iv) relatively low SEM (8.5–17.0 ms) and CV (3.6–6.4%) values with respect to absolute reliability, and (v) MDC95% values of 23.6 to 47.0 ms, which must be exceeded when using the electronic contact mat as a measuring device.

### 4.1. Concurrent Validity

Our findings regarding “excellent” correlations and rather low systematic bias values between the use of an electronic contact mat and a force plate for the analysis of the ground contact time during the execution of drop jumps indicate that the two measuring devices are in agreement. This finding supports the use of the contact mat for the valid assessment of the drop jump and is in accordance with the results of Rogan et al. [5] and in contrast to the results of Kenny et al. [4]. With regard to the latter, a possible reason is assumed to be differences in the methodological approach between the present study and the work of Kenny and colleagues. Specifically, we examined 79 subjects, and Kenny and co-workers only ten subjects, which limits the validity of their results. Further, and in contrast to the present study, the test persons had to perform countermovement jumps and squat jumps in addition to the drop jump assessment (requires stretching shortening cycle (SSC)). These types of jumps require either a comparatively long (countermovement jumps) or no (squat jump) SSC, and could therefore have had an influence on the proper execution of the drop jumps.

Our comparison of a contact mat with a light-barrier system (OptoGait^®^) also revealed “excellent” correlations, which also suggests that the two measurement devices are in agreement. However, the calculation of systematic bias values revealed larger deviations between these two devices than between the contact mat and the force plate. Therefore, the contact mat seems to be a better alternative as a criterion device for conducting low-cost drop jump analysis under field conditions.

### 4.2. Test–Retest Reliability

In the present study, independent of the drop height, “excellent” correlations and relatively low SEM and CV values were found for the electronic contact mat, as well as for the two criterion devices. This finding suggests that all three testing devices are consistent in repeated data collection, and thus provides evidence in favor of their reliable use for the analysis of the drop jump. This is in line with the results of previous studies [6,19,20]. For example, Markwick et al. [6] reported "excellent" correlations (ICC = 0.87–0.99) for the repeated recording (test distance: 1 minute) of the drop jump using an electronic contact mat from drop heights of 20, 30, 40, and 50 cm by young, physically active adults. In another study, Feldmann et al. [19] used a force plate to investigate 13 young, physically active adults who performed drop jumps from drop heights of 30 and 60 cm at intervals of 48 h. As a result, they found “excellent” correlations (ICC = 0.94), irrespective of the drop height. In another study by Byrne et al. [20], 19 athletes (age: 23.1 ± 2.9 years) performed drop jumps from four drop heights (30, 40, 50, 60 cm) at intervals of 48 h. The jumps were recorded with the OptoJump^®^ light-barrier system and resulted in “excellent” correlations (ICC = 0.81–0.87) regardless of the drop height. Accordingly, all three testing devices appear to be suitable for the reliable analysis of the ground contact time during the execution of drop jumps from different drop heights.

### 4.3. Minimal Detectable Change

From the MDC_95%_ values it can be deduced that, depending on the measuring device and jump condition, a change of 23.6 to 50.9 ms between repeated measurements can be attributed to measurement errors. In other words, only for values outside of this range can a change in the ground contact time be classified as relevant. There are several studies [21,22] that have shown training-related changes in ground contact time at this level. For example, Simpson et al. [21] investigated the wearing of weight vests on the jumping behavior of young adults. The participants in the intervention group wore the vests for 32 h a week in everyday life and three times a week during the training sessions. During the three-week training phase, and subsequent detraining phase (i.e., no wearing of the weight vest), there was a significant reduction in ground contact time in the intervention group of 72 ms during the execution of the drop jump (drop height: ~46 cm). In another study, Zemkova and Hamar [22] analyzed the impact of a combined agility and balance training (6 weeks, 4–5 times per week for 30 minutes) in young basketball players. The training group showed a significant reduction of the ground contact time of 33 ms as compared to the control group (only agility exercises) in performing drop jumps from a drop height of 45 cm.

## 5. Conclusions

The aim of the present study was to analyze the concurrent validity and the test–retest reliability of an electronic contact mat in comparison to a force plate (gold standard) and a further criterion device (OptoGait^®^ light-barrier system) for the assessment of ground contact time during the execution of drop jumps from different drop heights (24, 43, 62 cm) in physically active young adults. Regardless of the drop height, the results obtained demonstrate both a correspondence of the measuring devices (especially between the force plate and the contact mat) and a correspondence between repeated data collections (test–retest reliability). From this, it can be deduced that it is possible to carry out a valid, as well as reliable, analysis of the ground contact time during the execution of drop jumps from different drop heights in physically active young adults by means of an electronic contact mat. Thus, the contact mat used represents a cost-effective, transportable, and field-usable alternative for the investigation of the ground contact time during drop jumps. Depending on the jump condition when using the electronic contact mat, a value outside the range of 23.6 to 47.0 ms must be exceeded for repeated testing in order to obtain a practically relevant change.

## Figures and Tables

**Table 1:** Ground contact times (ms) achieved in the test and retest (1 week apart) by the measuring device.

Characteristic	Force Plate			OptoGait^®^			Contact Mat		
	Test	Retest	*p*-/*d*-Value	Test	Retest	*p*-/*d*-V	Test	Retest	*p*-/*d*-Value
Drop height: 24 cm									
two-legged	189.2 ± 29.3	189.5 ± 30.8	.91/.01	217.6 ± 30.9	219.8 ± 29.5	.41/.07	185.0 ± 27.6	186.6 ± 29.1	.53/.06
one-legged, left	241.0 ± 33.4	245.7 ± 30.9	.05/.15	280.7 ± 35.7	287.2 ± 34.1	.03/.19	237.4 ± 35.9	247.7 ± 30.3	.01/.31
one-legged, right	242.6 ± 36.4	249.5 ± 30.8	.08/.20	279.1 ± 30.9	288.9 ± 35.1	.01/.29	239.2 ± 27.0	250.6 ± 31.6	.01/.36
Drop height: 43 cm									
two-legged	201.4 ± 35.2	205.0 ± 30.0	.10/.10	231.7 ± 37.8	236.6 ± 32.5	.04/.13	200.4 ± 33.9	204.0 ± 28.5	.11/.11
Drop height: 62 cm									
two-legged	216.3 ± 33.6	227.3 ± 32.8	.01/.34	248.4 ± 37.3	256.1 ± 35.9	.05/.21	214.3 ± 33.5	226.8 ± 33.1	.01/.38

The data represent the mean value ± standard deviation. Cohen’s *d* [18] can be classified as being small (0 ≤ *d* ≤ 0.49), medium (0.50 ≤ *d* ≤ 0.79), or large (*d* ≥ 0.80).

**Table 2:** Results on the concurrent validity of the electronic contact mat as compared to the force plate and the OptoGait^®^ light-barrier system.

Characteristic	Contact Mat vs. Force Plate					Contact Mat vs. OptoGait^®^				
	ICC (95% CI)	Bias (ms)	LoA (ms)	Inter-Cept	Slope	ICC (95% CI)	Bias (ms)	LoA (ms)	Inter-Cept	Slope
Drop height: 24 cm										
two-legged	0.98 (0.97–0.99)	3.1	−7.2 ± 13.4	−0.007 (0.104)	1.052 (<0.01)	0.95 (0.93–0.97)	31.4	13.1 ± 49.8	0.020 (0.006)	1.062 (<0.01)
one-legged, left	0.96 (0.94–0.97)	−0.1	−19.7 ± 19.5	0.013 (0.087)	0.947 (<0.01)	0.95 (0.93–0.97)	41.7	22.6 ± 60.8	0.033 (<0.01)	1.034 (<0.01)
one-legged, right	0.95 (0.93–0.97)	−0.6	−17.7 ± 16.5	−0.002 (0.809)	1.009 (<0.01)	0.97 (0.95–0.98)	39.2	24.4 ± 54.1	0.013 (0.047)	1.107 (<0.01)
Drop height: 43 cm										
two-legged	0.99 (0.98–0.99)	−0.4	−10.7 ± 10.0	−0.006 (0.137)	1.027 (<0.01)	0.98 (0.97–0.99)	31.4	19.2 ± 43.7	0.012 (0.003)	1.100 (<0.01)
Drop height: 62 cm										
two-legged	0.99 (0.98–0.99)	−0.4	−10.3 ± 9.5	−0.004 (0.343)	1.016 (<0.01)	0.98 (0.96–0.99)	32.6	18.8 ± 46.4	0.017 (0.002)	1.072 (<0.01)

CI is confidence interval; ICC_2.1_ is intra-class correlation coefficient; LoA is limits of agreement.

**Table 3:** Results on relative and absolute test–retest reliability by measuring instrument.

Characteristic	Force Plate				OptoGait^®^				Contact Mat			
	ICC (95% CI)	SEM (ms)	CV (%)	р_c_	ICC (95% CI)	SEM (ms)	CV (%)	р_c_	ICC (95% CI)	SEM (ms)	CV (%)	р_c_
Drop height: 24 cm												
two-legged	0.81 (0.68–0.89)	13.0	5.3	0.81	0.82 (0.70–0.90)	12.6	4.6	0.82	0.83 (0.71–0.90)	11.8	5.1	0.82
one-legged, left	0.88 (0.78–0.93)	11.3	3.6	0.87	0.84 (0.72–0.91)	14.1	3.8	0.82	0.74 (0.58–0.85)	17.0	5.1	0.71
one-legged, right	0.70 (0.52–0.82)	18.4	4.9	0.69	0.90 (0.82–0.94)	10.6	3.7	0.86	0.92 (0.86–0.95)	8.5	3.9	0.85
Drop height: 43 cm												
two-legged	0.90 (0.83–0.94)	10.3	4.3	0.90	0.90 (0.83–0.94)	11.1	4.0	0.89	0.89 (0.80–0.94)	10.5	4.3	0.88
Drop height: 62 cm												
two-legged	0.77 (0.62–0.87)	16.1	6.3	0.73	0.76 (0.60–0.86)	18.1	5.5	0.74	0.75 (0.59–0.85)	16.9	6.4	0.70

CI is confidence interval; CV is coefficient of variation; ICC_3.1_ is intra-class correlation coefficient; SEM is standard measurement error; р_c_ is Lin´s concordance correlation coefficient.

**Table 4:** Minimal detectable change (MDC95% in ms) results by measuring instrument.

Characteristic	Force Plate	OptoGait^®^	Contact Mat
Drop height: 24 cm			
two-legged	36.0	34.9	32.7
one-legged, left	31.4	39.1	47.0
one-legged, right	50.9	29.4	23.6
Drop height: 43 cm			
two-legged	28.4	30.6	29.1
Drop height: 62 cm			
two-legged	44.6	50.1	46.9
